# Metabolomics as a Tool to Elucidate the Sensory, Nutritional and Safety Quality of Wheat Bread—A Review

**DOI:** 10.3390/ijms22168945

**Published:** 2021-08-19

**Authors:** Adriana Păucean, Vlad Mureșan, Simona Maria-Man, Maria Simona Chiș, Andruța Elena Mureșan, Larisa Rebeca Șerban, Anamaria Pop, Sevastița Muste

**Affiliations:** Department of Food Engineering, Faculty of Food Sciences and Technology, University of Agricultural Sciences and Veterinary Medicine of Cluj-Napoca, 3-5 Mănăştur Street, 400372 Cluj-Napoca, Romania; adriana.paucean@usamvcluj.ro (A.P.); simona.chis@usamvcluj.ro (M.S.C.); andruta.muresan@usamvcluj.ro (A.E.M.); larisa-rebeca.serban@usamvcluj.ro (L.R.Ș.); anamaria.pop@usamvcluj.ro (A.P.); sevastita.muste@usamvcluj.ro (S.M.)

**Keywords:** metabolite profiling, omics, wheat breeding, dough fermentation, bakery product quality

## Abstract

Wheat (*Triticum aestivum*) is one of the most extensively cultivated and used staple crops in human nutrition, while wheat bread is annually consumed in more than nine billion kilograms over the world. Consumers’ purchase decisions on wheat bread are largely influenced by its nutritional and sensorial characteristics. In the last decades, metabolomics is considered an effective tool for elucidating the information on metabolites; however, the deep investigations on metabolites still remain a difficult and longtime action. This review gives emphasis on the achievements in wheat bread metabolomics by highlighting targeted and untargeted analyses used in this field. The metabolomics approaches are discussed in terms of quality, processing and safety of wheat and bread, while the molecular mechanisms involved in the sensorial and nutritional characteristics of wheat bread are pointed out. These aspects are of crucial importance in the context of new consumers’ demands on healthy bakery products rich in bioactive compounds but, equally, with good sensorial acceptance. Moreover, metabolomics is a potential tool for assessing the changes in nutrient composition from breeding to processing, while monitoring and understanding the transformations of metabolites with bioactive properties, as well as the formation of compounds like toxins during wheat storage.

## 1. Introduction

Worldwide, bread and bakery products represent the essential staple food of the human diet. For over two decades, the social habits of modern consumers have been oriented towards convenient food products due to the lack of time for cooking, shopping, or food preparation [1]. With respect to bread and bakery product innovation and consumption, it is considered that pleasure and health are also important trend drivers [2]. Consumers’ purchase decisions are largely influenced by the health trend. Bakery products seen as healthy are usually purchased. In this context, the new trends in bread making are guided by two strategic factors: (a) the development of innovative products with functional ingredients rich in bioactive compounds, which are able to satisfy new consumers’ requirements related to a healthy diet and (b) the utilization of conventional and innovative processing and preservation techniques in order to improve the product quality [3].

Starting with the 21st century, the achievements obtained at the level of analytical tools and methods are unprecedented. Moreover, the boundaries between the fields of research are intertwined and consequently, lead to emerging interdisciplinary research areas such as food and health. In this context, the new term Foodomics was proposed as “*a discipline that studies the food and nutrition domains through the application of advanced omics technologies to improve consumer’s well-being, health, and confidence*” [4]. For this goal, foodomics analyzes the groups of compounds existent in a “*food sample or biological system interacting with the investigated food at a given time*”, namely the *foodome*. *Foodome* characterization is obtained by using instrumental omics platform (transcriptomics, proteomics and metabolomics) and by performing multidisciplinary approaches such as nutrigenomics, nutrigenetics, microbiomics, toxicogenomics, nutritranscriptomics, nutriproteomics, nutrimetabolomics, etc. Covering a large area of interest, foodomics is used in research related to food bioactivity and health, food quality and traceability, food safety and provides valuable results in breeding, post-harvesting changes control, functional foods and nutraceuticals [5]. As an emerging field within “omics” sciences, metabolomics is aiming to determine and quantitatively analyze the small metabolites (small-molecules) from the system, allowing for a comprehensive overview of biological systems or other complex materials [6]. The metabolome represents all of the low molecular weight (<1500 Da) compounds present in a cell, tissue, or organism at a specific time [7]. Metabolomics is an advantageous approach that could give a deep insight into food characteristics but due to the diversity within the metabolome, an immense analytical challenge exists [8]. Several stages such as sample preparation, metabolite extraction, derivatization, metabolite separation, detection and data treatment are required in metabolomics analyses, even if some of them are optional. Due to the very complex content of food matrices with compounds that have different physical and chemical properties, appropriate sample preparation is of crucial importance during analysis and interpretation to avoid artifacts. Solid samples are generally ground under liquid nitrogen or after freeze-drying, while sometimes, antioxidant reagent such as butylated hydroxytoluene is added to preserve metabolites sensitive to oxidation. Other homogenization techniques such as electric grinder, tissue lyser and ultrasonic are also used to enhance the release of metabolites. Sample drying is often used to avoid enzyme-mediated reactions that cause metabolite changes, but from all the drying methods, freeze-drying is preferred, as it enables maintaining the cold chain [9]. Even if modern techniques like microwave-assisted extraction and supercritical fluid extraction can be used for metabolite isolation, their impact on compound stability is high. Thus, solvent-based extraction is widely used. Chloroform, methyl tert-butyl ether, a mixture of methanol and water (1:2, *v/v*) or methanol/water/formic acid (70:30:0.1) are indicated for hydrophilic and hydrophobic metabolites extraction. When all metabolites are extracted in one step, the mixture of methanol/chloroform and water is the popular one, with the ratio of solvents determining the proportion of polar vs. non-polar metabolites [10]. However, separation and detection are considered key stages and the most used techniques for separation are liquid chromatography (LC) including high performance (HPLC) and ultra-performance (UPLC), gas chromatography (GC), electrophoresis and capillary electrophoresis (CE) often coupled with mass spectrometry (MS), nuclear magnetic resonance (NMR), and near infrared (NIR) spectrometry. For metabolite detection, frequently ultraviolet (UV), NIR, Fourier transform infra-red spectroscopy (FTIR), MS, or NMR techniques are used [10]. Due to the extremely large number and complexity of metabolomics, data processing, and pre-treatment (peak alignment, normalization, matching, etc.) are compulsory to identify between the significant metabolites [5]. Subsequent multivariate data analysis is run using two types of models. Firstly, unsupervised models including principal component analysis (PCA), cluster analysis (HCA), nonlinear mapping (NLM) are performed to detect the clustering of data; secondary, supervisory models (partial least square discriminant analysis (PLS-DA); linear discriminant analysis (LDA); orthogonal partial least square discriminant analysis (OPLS-DA)) are applied to discriminate the metabolites [5].

Targeted and non-targeted analyses are known as the main types of metabolomics approaches. Targeted analyses are focused on the identification and quantification of specific groups of metabolites and generally assess the modifications of these compounds under selected conditions. On the contrary, non-targeted approaches aim to determine as many metabolites as possible without compulsory identification or quantification. Another classification of the metabolomics approaches is related to the defined goal of the study and from this point of view, metabolomics could be discriminative, informative, and/or predictive. The differences between the samples are determined by discriminative analyses and multivariate data analyses in order to obtain clear results. Informative metabolomics analyses are run to give inherent information (biomarkers, pathways, novel bioactive compounds, etc.) about the metabolites and, in this way, metabolite databases are developed. When it comes to predictive metabolomics approaches, statistical models on metabolite profiles are created to predict variables that are difficult to determine otherwise [10].

With respect to compounds annotation and identification, metabolomics studies depend on confidence levels, which can vary from unknown (level 4) to identified metabolites (level 1) with putatively characterized/annotated compounds as intermediate levels (2,3), as it was recognized by the international Metabolomics Standards Initiative (MSI) [11]. In order to increase the confidence level in metabolite annotation, molecular networking tools are being developed and web platforms with large types of supported software allow compounds annotation and identification. However, due to the extremely large number of compounds, the full annotation requires complementary tools. Moreover, bioinformatics researchers’ efforts helped to solve issues in some fields (e.g., proteomics) but they are still making efforts in the field of small molecules that need structure clarification [9,12].

Recent research articles highlighted the effectiveness of metabolomics as a tool to monitor and predict the quality, processing and safety of raw materials and final products, aiming to improve the consumer’s health and confidence [7,13,14,15].

**Figure 1:**
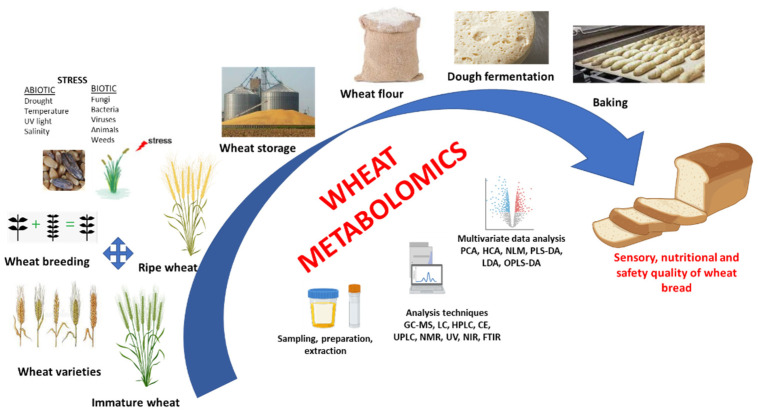
Metabolomics as a tool to elucidate the sensory, nutritional and safety quality of wheat bread.

Within the research area of wheat and breadmaking, metabolomics was used generally with the specific goal of quality improvement (Figure 1). Therefore, numerous studies reported the metabolite profiling of different types of bread made by preferment or sourdough technology [16,17,18,19]. Furthermore, during the last decade, the performed metabolomics approaches tried to provide new insight into the relationship between the raw materials, dough fermentation, aroma profile, nutritional value and bread quality. In this regard, metabolite profiling is considered to be an efficient investigation tool for cereal responses to abiotic and biotic stresses as well as to determine the chemical compositions of the kernel and the main cereal products. The classification of flours according to their geographical origin was proposed also by Brescia et al. [20], using a metabolomics approach. Flours and breads obtained from different types of common and durum wheat were clearly separated and furthermore, the flours were classified according to their area of production, using ^1^H NMR-MAS NMR (proton high-resolution magic angle spinning nuclear magnetic resonance) and isotopic investigation. Even though the dough fermentation process is crucial for the flavor and loaf volume, the regulation of this process is one of the most difficult technological steps in bread production. Thus, metabolomics was applied to study the metabolite profile in fermented dough and to measure the biochemical profiling and metabolite modifications during the fermentation process. The bread flavor is related to the volatile derivatives and their composition is greatly influenced, among other factors, by mixing stage, fermentation conditions (time, temperature), type and amount of fermenting microorganisms (yeast and/or lactobacilli), baking conditions. Volatile metabolites in fermented bakery products were elucidated by metabolomics analysis in order to understand the role of flour and fermentation process in increasing rheological and volatile metabolites [21,22]. With regard to wheat metabolite profiling, the identification of metabolites with modified levels as a result of exposure to abiotic stressors is usually addressed. Targeted approaches are used sometimes but as the majority of studies show, non-targeted approaches could lead to better identification of known or unknown metabolites. Generally, the most common approach for wheat profiling is the high-resolution mass spectrometry technique. In the case of wheat sourdough bread, targeted analyses are mainly preferred to monitor compound traceability from substrates to sourdough or the production of new ones [9,23].

## 2. Metabolomics Approaches for Wheat Production and Processing

It is now well known that metabolomics can lead to finding solutions for high-quality food production and consumption. Recent studies have already indicated the potential of metabolomics for the “*from the farm to the fork*” concept due to its capacity to identify and quantify major chemical compounds as well as secondary metabolites. Metabolomics approaches via metabolite studies can establish a relation between wheat and environment, between the crop, soil and other ecosystem aspects. As a result, different correlations could be identified between the quality characteristics of bread (nutritional, organoleptic features) and the investigated metabolites [7].

Villate et al. [9] reported in a recent review that the analysis of the plant metabolome can give a whole picture of the plant’s physiological state, helping the breeders to develop stress-resistant plants. Trait-specific markers could be identified by metabolomics approaches and thus the selection of improved breeding materials is facilitated. Due to environmental stress, plants produce primary and secondary metabolites aiming at their defense. Synthesize pathways models of these metabolites during the presence of various abiotic stresses were recently proposed by Singh et al. [24]. In this specific context of metabolomics as a prediction tool for plants performance under environmental stress, the build and use of the prediction models is considered to be an important objective.

Cereals and especially wheat are plants of crucial importance for human nutrition, providing the body with essential nutrients such as carbohydrates, proteins, dietary fibers, vitamins and phenolic compounds, flavonoids, carotenoids and fructans [13]. A large part of these metabolites is recognized as biologically active compounds due to their demonstrated protection effect against cardiovascular disorders, type 2 diabetes, some cancers, etc. As a result, an increase in the consumption of cereals, especially whole grains was recorded all over the world.

In spite of this worldwide spread of cultivation and use of wheat as a food and feed raw material, less than 30% of the total studies of plant metabolomics report it [7]. There is general concern about wheat yield and quality in the context of global climate change and the repeated exposure to prolonged drought periods. Using metabolomics approaches different aspects of the biological and physiological changes caused by abiotic factors and plant responses to these factors were investigated.

Several analytical techniques were used for metabolomics studies in different sample parts (leaf, mature and immature grain, root, shoot, seedling, spikelet, stem, wholemeal flour) of *Triticum* species under abiotic stress, including GC-MS [25,26,27,28,29,30,31,32]; LC-MS [33,34,35,36,37]; UPLC or UHPLC-qTOF [31,38]; LC-HRMS and Thermo Q-Exactive Oribtrap [39]; GC-qTOF/LC-qTOF [40]; GC-QTOF [15]; GC–TOF–MS [41]; LC-MS/MS and UHPLC-MS/MS [42] ^1^H-NMR [43].

Metabolomics analyses applied to wheat organ samples consist of stage successions, which include sample preparation, metabolite extraction, possible derivatization and separation, detection and data treatment. Generally, considering the physicochemical properties of the metabolites, liquid chromatography-mass spectrometry (LC–MS) is mostly used for phytohormones, amino acids, oligosaccharides identification. Also, gas chromatography coupled to mass spectrometry (GC–MS) is used to identify primary metabolites (after derivatization if non-volatile compounds are investigated). Nuclear magnetic resonance (NMR) is used as a powerful tool in metabolites with small molecular weight (<50 kDa) detection and is based upon the utilization of magnetic properties of nuclei of atoms under a magnetic field [7]. In order to draw inferences about the molecular formula of unknown compounds, HRMS detectors (TOF-time of flight, FT-ICR- Fourier Transform Ion Cyclotron Resonance or Orbitrap mass spectrometers) are used. The MS/MS spectra obtained in hybrid mass spectrometers (qTOF, qOrbitrap) provide structural information about the unidentified metabolites [9]. Thus, the most used techniques are: *GC–MS*: Gas chromatography mass spectrometry; GC-TOF: Gas chromatography coupled with time of flight mass spectrometry; GC–TOF–MS Gas chromatography time-of-flight mass spectrometry; RPLC-qOrbitrap: Reversed phase liquid chromatography coupled with quadrupole Orbitrap tandem mass spectrometry; RPLC-qTOF: Reversed phase liquid chromatography coupled with quadrupole time of flight tandem mass spectrometry; LC-qTOF: Liquid chromatography coupled with quadrupole time of flight tandem mass spectrometry; ^1^H-NMR: Proton nuclear magnetic resonance; LC-MS: Liquid Chromatography coupled with Mass Spectrometry; UHPLC-MS/MS: Ultra-high performance liquid chromatography tandem mass spectrometry.

Metabolomics studies on wheat responses to stress reported that several compounds are involved in wheat tolerance to drought, such as proline, glycine betaine, γ-aminobutyric acid (GABA), β-aminobutyric acid, 5-aminolevulinic acid, and some polyamines that compete with ammonium conversion in plants, especially putrescine and the raffinose family oligosaccharides. It was also found that protein synthesis and free amino acid accumulation (excepting the glutamate) and carbohydrate content can be affected by osmotic stress [7]. Several studies reported that the metabolomics responses to drought stress varied in magnitude between wheat genotypes. Variations were identified for amino acids, polyols, sugars, fatty acids, organic acids [42]. With respect to temperature response, sugar accumulation and abscisic acid biosynthesis were mainly reported as plant responses [44]. Heat stress caused malondialdehyde and proline increment and lowered total carotenoid content, producing changes in lipid composition [32,45]. Regarding the suboptimal N or S necessities, it was reported that it is affecting the leaf metabolite compositions more than the wheat grain.

The resistance of wheat to *Fusarium graminearum* was also pointed out by metabolomics studies since Fusarium head blight (FHB) is one of the main fungal diseases of wheat crops. The wheat resistance responses were related to hydroxycinnamic acid amides, phenolic glucosides, and flavonoids biosynthesis. In addition, metabolomics approaches provided deeper results and it was reported that the metabolites synthetized in FHB resistance were hydroxycinnamic acid amides, especially coumaroyl-agmatine and coumaroyl-putrescine. When *Zymoseptoria tritici* attack on wheat was studied, a reduction in fatty acids and some sugars was found as an indirect response. The presence of resistance genes in the wheat D genome confers some protection but also evolutionary selection and breeding were found as important factors in resistance responses along with the type of pathogen, the moment of sampling, and the growing stage [7]. The influence of the beneficial bacteria associated with roots on wheat metabolome revealed significant changes in biomass and nutritional uptake. It was reported that N_2_-fixing bacteria (*Azospirillum* spp., *Azorhizobium* spp.) increased the N uptake and chlorophyll content in the case of wheat [46].

Metabolomics approaches could be used successfully in wheat quality control since they could provide specific metabolites which differ between species. So, alkylresorcinols, phosphatidylcholines, lysophosphatidylcholines, triacylglycerols, and galactolipids were found as specific metabolites by several studies which analyzed *Triticum turgidum* var. *dicoccum* L.(emmer), *Triticum monococcum* L. (einkorn), *Triticum spelta* L.(spelt), *Triticum durum, Triticum aestivum* L. species [33,37,47].

When wheat quality is discussed, two types of criteria are analyzed depending on the farmer point of view or the processor one. The first one considers wheat quality in terms of yield and resistance to stress conditions, while for the miller wheat quality is expressed by the content and the quality of protein, flour yield and baking performance [48]. The protein and moisture contents along with the safety properties regarding the mycotoxin amount are widely used for wheat commercialization. Recently, Longin et al. [15] demonstrated by metabolomics approaches that bread aroma could be used as a new target criterion into the wheat product chain by analyzing 18 old wheat varieties (1962–1999) and 22 modern varieties (2005–2014). The authors stated that the variety and the cultivation environment significantly influenced bread aroma. Also, they found that bread aroma was not significantly correlated with parameters like yield or bread volume and concluded that exists wheat varieties with high yield, high breadmaking quality and intensive bread aroma. These results could be used in predictive breeding by combining the high breadmaking quality with aroma markers and metabolite profile of the wheat flour.

## 3. Metabolite Profiling during Wheat Dough Fermentation

Fermentation is one of the most important stages in breadmaking technology and has a large influence on bread quality. During the stage of dough development, the key transformation is related to the ability of the dough matrix to retain the carbon dioxide gas which is generated by yeasted preferment or by the combined fermentation of lactic acid bacteria and yeast. Moreover, the preferment or sourdough method will significantly influence the flavor of the bread. Compared to yeasted bread, sourdough breads have a specific acidic flavor mainly due to the ratio of acetic to lactic acid flavor notes [49].

Sourdough fermentation is considered to be a key process during breadmaking due to its crucial influence on the bread’s nutritional and organoleptic characteristics. Nowadays, a large body of literature highlights dough fermentation as a powerful tool to improve the bioactive compound content as well as the aroma, taste and texture of the final product [50,51,52,53,54,55]. The fermentative activity of lactic acid bacteria (LAB) and/or yeasts in dough leads to an enhanced content of health-promoting compounds which is one of the consumers’ specific requirements. However, even though an increased demand for bakery products with functional properties is noticed, the consumption patterns of new products are strongly determined by their sensorial characteristics [13]. Thus, metabolite profiling is performed to monitor metabolite changes during fermentation and to predict the sensorial and nutritional quality of baked products [14].

Sourdough, the mixture of flour and water fermented by microorganisms (LAB and yeasts) which derive from the flour or from a starter culture, has several uses in breadmaking such as leavening agent, acidifier or flavor carrier. Yeasts are mainly responsible for the dough leavening and the formation of the aroma compounds, while LAB generates aromatic derivatives in smaller amounts but produces acidification. However, it is now known that during sourdough fermentation the metabolic interactions which occur between LAB and yeasts lead to the specific flavor characteristics. The most relevant LAB species belong to *Lactobacillus, Leuconostoc, Weissella*, whereas the yeast species are *Saccharomyces cerevisiae* and *Candida* sp. [23].

The bread flavor is a result of several factors such as the aroma compounds naturally existing in the flour and the interactions between the indigenous and exogenous enzymes during fermentation. Moreover, the specific metabolic pathways of LAB (homo/heterofermentative) or yeast lead to different volatiles in the fermented dough. Thus, the volatile profile is significantly influenced by the microbial species as well as the strains of the same species. One of the major aroma precursor groups are free amino acids, which are released by LAB and yeasts. During fermentation, amino acids are transformed via the Ehrlich pathway to aldehydes or the corresponding alcohols [56]. Generally, fermentation with *S. cerevisiae* and other yeasts (e.g., *Candida, Pichia*) led to a higher concentration of alcohols, esters and carbonyl compounds. Among the key compounds are ethanol, methylpropanol, 2- and 3-methylbutanol, ethyl acetate and diacetyl (2,3-Butanedione) [56]. When LABs are the fermenting agents, lactic and acetic acids are important factors in dough and bread aroma, but also volatiles like ethyl acetate, hexyl acetate are formed. Another group of volatiles formed during LAB metabolism is that of carbonyl compounds. These compounds may derive from flour’s lipoxygenases activity on unsaturated fatty acids present in the flour or as a specific metabolic pathway of homofermentative species, as in the case of diacetyl and hexanal. Also, nonanal is the main oxidation product of oleic acid and is widely found in wheat sourdough. Benzaldehyde, which is an aromatic aldehyde, results from autoxidation of 2,4-decadienal or from aromatic amino acid degradation. Ketones, like benzophenone and acetoin, could be identified in the early stage of sourdough fermentation and are indicators of bread freshness [57].

Most studies applied a targeted metabolite analysis in order to determine the traceability of the specific metabolites (carbohydrates, organic acids, amino acids, etc.) from the substrates to fermented dough. (HS-)SPME-GC–MS analysis (headspace-solid-phase microextraction in combination with gas chromatography coupled to mass spectrometry) is usually used for the determination of volatile metabolites from sourdough, while for the residual compounds LC-MS or with other detection methods are preferred [23]. Metabolomics profiling at each stage of dough fermentation is an important approach to understand when different metabolites are produced [58]. Metabolomics studies revealed that to obtain bakery products with improved functional and sensorial properties by sourdough technology, the interaction between the strain type and the substrate composition is a determining factor. During the sourdough fermentation, lipids, proteins, carbohydrates, phenolic acids and folates are transformed as a result of microbial metabolism, contributing to bread quality in terms of texture, volume, shelf-life, flavor and nutritional quality. Thus, dietary fibers, prebiotic oligosaccharides, bioactive peptides and amino acids enhance the bread’s functional properties. Moreover, some antinutritive factors, such as phytic acid, are eliminated and consequently the bioavailability of minerals is increased [50]. This is a result of pH decreases during sourdough fermentation which causes the increment of endogenous phytase which becomes more effective than the microbial one. On the other hand, pH reduction contributes to the increment of phenolic acids, total phenolic compounds, and alkylresorcinols amounts [52]. The metabolomic and antioxidant shifts monitored by ^1^H NMR spectroscopy during spelt sourdough fermentation revealed that microbial proteolysis released bioavailable branched amino acids (BCAA) which may lead to the production of bioactive peptides. The bioactive peptides have antioxidant and anti-inflammatory activity and antihypertensive effects [52,59]. The antioxidant activity of sourdough is also enhanced by free amino acids, organic acids and aromatic compounds, the latter being related also to the protein enzymatic depletion. For example, it was previously reported that phenylalanine is a precursor of plant phenolic compounds [60] and now it is considered that amino acids could influence directly the increment of phenolic compounds [59]. In recent years, attention was paid to γ-aminobutyric acid (GABA), a non-protein amino acid with numerous health benefits like neurotransmitter, hypotensive, diuretic, tranquilizing effects, inhibits leukemia cell proliferation, stimulates cancer cell apoptosis [61,62]. GABA is formed from glutamic acid via enzymatic reaction and it is naturally present in grain or it could be produced by LAB and/or yeast activity [52,63]. Sourdough fermentation is considered a powerful tool to enhance the bioavailability of minerals due to the phytase activity which could hydrolase the phytates and release minerals (Ca, Fe, K, Mg, Mn, Zn). The reduction of phytate content was effective in the case of *Lactobacillus* ssp. and certain yeasts (*S. cerevisiae*, *Kluyveromyces marxianus*) as well as by co-cultivating LAB and yeasts [64,65]. The impact of fermentation conditions on the amount and the bioavailability of beta-glucans, fructans, resistant starch and arabinoxylans, as the most prominent dietary fibers from KAMUT^®^ Khorasan and durum wheat, was studied by Saa et al. [14]. Two types of fermentation were conducted, a sourdough fermentation with *Lactobacillus* ssp. and *S. cerevisiae* and an industrial one with only *S. cerevisiae* as a microbial agent. Results indicated that KAMUT^®^ Khorasan bread fermented only with *S. cerevisiae* had higher content of fructans and resistant starch. The sourdough fermentation decreased the beta-glucans amount but led to an increased amount of arabinoxylans.

Several metabolomics studies indicated that different substrates did not lead to the same metabolic profile in sourdough after fermentation [13,66,67]. Ferri et al. [13] studied *L. plantarum* fermentation in durum and KAMUT^®^ Khorasan wheat flours and reported a higher metabolic diversity in durum sourdough. Ten strains of *L. plantarum* were used in the study and results showed that the metabolic pattern was directly correlated to the inoculated strains. The antioxidant activity and the polyphenol content are the most important indicators for the potential of *L. plantarum* to enhance the nutritional and sensorial characteristics of the sourdough. Correlations were reported between volatile molecules and polyphenols, as well as between hydrocarbons, esters and antioxidant capacity. However, due to the strain/substrate interactions, differences between the correlated metabolites were found for the tested flours.

The influence of the substrate composition was highlighted by Pizarro and Franco [57] when they analyzed the volatile compounds from whole wheat and all-purpose sourdough during 28 days of fermentation. Whole wheat sourdough was more effective in aroma compounds production with key compounds like hydrocarbons, heterocyclic compounds (furans, pyrrole, and pyrazole), 2-methyl-1-butanol, 3-hydroxy-2-butanone, 2-pentadecanone, benzophenone, and benzaldehyde. In the case of all-purpose sourdough, aldehydes were identified to a higher extent but also benzophenone, 2-pentadecanone, nonanal, and benzothiazole were specific compounds.

A non-targeted metabolomics approach was applied by Koistinen et al. [67] providing a broad comprehension of the sourdough metabolite profile. Metabolic profiling utilizing LC–QTOF–MS was carried out on whole-grain wheat and rye bread, prepared with baker’s yeast or a sourdough starter containing *Candida milleri*, *Lactobacillus brevis* and *Lactobacillus plantarum*. The results clearly indicated that fermentation causes changes in metabolites profile leading to bioactive compounds with health beneficial effects, especially in whole-grain wheat and rye. Sourdough fermentation significantly increased the amounts of branched-chain amino acids (BCAAs) leucine and isoleucine and several small peptides containing BCAAs, especially in rye sourdough due to an intensive proteolytic activity. BCAAs were correlated with lower insulin response [68,69] while small peptides are considered compounds with antioxidant and antihypertensive activity [70]. As a result of microorganism metabolism, phenolic acids were transformed into dihydroferulic acid, dihydrocaffeic acid and dihydrosinapic acid, feruloylagmatine and p-coumaroylputrescine, compounds known with antioxidative and antimicrobial effects [67,71].

The specific interaction between LAB and the flour type was pointed out by Galli et al. [72] analyzing total phenolic acids and the antioxidant activity of a blue-grained wheat variety rich in anthocyanins and a conventional red-grained variety. It was found that sourdough prepared with a variety rich in anthocyanins and certain *Lactobacillus* ssp. exhibited higher antioxidant activity and phenolic acids amount, highlighting that substrate composition is essential for bread pro-health value.

In the same regard, Saa et al. [14] used E-nose and SPME-GC–MS and reported the capacity of this approach to discriminate between different types of flour (KAMUT^®^ khorasan and durum wheat) and also to distinguish samples at different maturation stages (milky and full ripe). *Lactobacillus plantarum* 98a, *Lactobacillus sanfranciscensis* BB12, *Lactobacillus brevis* 3BHI and *Saccharomyces cerevisiae* LBS strains were used for flours inoculation. It was clearly demonstrated that different flours produce different volatile profiles of sourdough as a result of the interaction between the LAB strain and the flour composition. Therefore, due to the higher protein content of KAMUT^®^ Khorasan flour a wider range of sulfur compounds was identified in the related sourdough [21]. The KAMUT^®^ Khorasan sourdough and fermented breads were characterized by higher content of volatile compounds like acetic acid, pentanoic acid, hexanol, ethanol, heptanal, hexanal and decanal. The higher amount of acetic acid found in this sourdough was due to the interactions between the substrate composition in nutrients and the LAB strains. Heptanal, hexanal and decanal were produced mainly due to the oxidative degradation of unsaturated fatty acids. The influence of the strain type could be highlighted in the case of ethanol and ethyl acetate production which were correlated with *L. sanfranciscensis* metabolism [14,21]. These studies emphasize the potential of SPME to detect specific volatile compounds from the sourdough matrices and the role of acidification, which induces several changes in flour composition and allows robust discrimination between groups. E-nose is considered a powerful tool to distinguish the cereal samples from the others and to discriminate the fermented and not fermented doughs [14,21].

SPME-GC/MS was used to determine the volatilome from sourdoughs made by inoculating wheat, quinoa and Kamut^®^ flours with *Lactobacillus paracasei*, *Lactobacillus plantarum* and *Lactobacillus brevis* strains. The volatile profile of the sourdough samples fermented for 48 h revealed significant differences within the classes of aldehydes, sulfur compounds, ketones, esters and acetates, alcohols, furans, pyrazines and acids. The volatile compounds formation was to a higher extent in Kamut and quinoa sourdoughs compared to wheat doughs which were characterized by a weak aroma. The use of composite flours made of wheat and Kamut and/or quinoa flours could lead to bakery products with an enhanced aroma profile. Quinoa sourdough is characterized by specific nutty, roasted and buttery notes, while Kamut sourdough revealed fruity, rose, green and sweet notes. When Kamut flour was inoculated with *L. plantarum* M4 and *L. paracasei* I1, high amounts of pentanal, hexanal, trans (E)-2-heptanal, nonanal, 2-octenal, 2,4-nonadienal and 2,4-decadienal were detected. Dimethylsulfide from the sulfur compounds group was found in the sourdough containing quinoa flour. Also, ketones, diacetyl and acetoin were quantified in higher concentrations in quinoa sourdoughs, especially when *L. paracasei* I1 and *L. brevis* T4 were the fermenting strains. These compounds are correlated with the amino acid content of quinoa flour, well known as a rich source of protein that could release free amino acids under the proteolytic activity of LAB [73].

The fermentation produced by bakery yeast (*Saccharomyces cerevisiae*) is of crucial importance in bread quality due to its influence on the flavor and loaf volume. The factors which are influencing the yeast metabolism during fermentation are temperature and time, sugar content, the type of strain and the cell concentrations [74]. Nakamura, Tomita and Saito [58] analyzed the metabolites formed in yeast fermented dough at each stage of fermentation and demonstrated that the metabolites variation is significantly influenced by the fermentation length and the yeast amount. Using metabolic approaches, several metabolites were significantly correlated with the bread flavor and volume. It is now well known that ethanol is the main compound produced by yeast; however, some authors also reported that as the dissolved oxygen content decreases due to its consumption, the succinic acid is formed. A higher amount of yeast speeds up the formation of ethanol and succinic acid, compounds able to enhance the dough development. Moreover, by increasing the fermentation time, glycerol content was noteworthy, showing influence on the dough gas retention. The metabolites which were found significantly correlated with the bread volume were γ-nonalactone, (E,E)-2,4-nonadienal and citronellal. These results were obtained by OPLS regression, and the authors consider that these metabolites may be criteria for the progress of fermentation even if they do not affect the bread swelling.

Yan et al. [75] used SPME-GC-MS and E-nose to determine key aroma compounds in Chinese steamed bread (CSB) produced with type I sourdough and baker’s yeast. This metabolic approach revealed ethyl acetate, ethyl lactate, hexyl acetate, (E)-2-nonenal and 2-pentylfuran as key volatile compounds from CBS produced with type I sourdough as compared to yeasted bread. Ethyl acetate is an ester formed from acetic acid and ethanol. The content of ethyl acetate was high in the proof dough and decreased after steaming. Ethyl lactate and hexyl acetate were formed during the steaming process as a result of esterification between acids and alcohols, a reaction accelerated by the high temperature. The 2-pentylfuran was found in low content in the proof dough, but its concentration increased after steaming. In the case of the bread fermented with *Saccharomyces cerevisiae* baker’s yeast, higher amounts of 3-methyl-1-butanol and phenylethyl alcohol were found. The 3-methylbutanol is formed during dough fermentation with yeast from leucine, which is a result of gluten decomposition by aspartic proteases. *S. cerevisiae* could transform amino acids in alcohols through the Ehrlich pathway [76].

The potential of metabolomics approaches in breadmaking is emphasized by several recent studies which focused on the metabolite modifications during the processing of fortified bread. These findings could be considered a step forward for a better understanding of how functional wheat substituents could impact human body health status. The results of these studies confirm that metabolomics approaches can indicate clear differences between flours, doughs and bread samples when wheat flour is substituted with functional flour or extracts. Non-targeted metabolomics is a potential tool to identify food markers when coupled to efficient data analysis [67]. Thereby, these markers could discriminate between flours if they possess unique secondary metabolites like sesamol in sesame seeds or rosmarinic acid in chia seeds. Also, the presence of these seeds in bakery products could be revealed by specific markers [77]. A very recent study performed a metabolomics approach to assess the impact of grape pomace powder on phenolic bioaccessibility and starch digestibility of wheat bread. This approach allowed the advanced characterization of grape pomace but more than that, the fortified bread samples profiling revealed the anthocyanins variation in relation to the bioaccessibility, starch hydrolysis and glycemic index. Moreover, according to the authors, these findings showed that grape pomace enriched bread could promote an antioxidant environment in the digestive tract [78]. In the same regard, Taglieri et al. [79] showed that secondary metabolites from Vitellote potatoes significantly improved the phenolic content and antioxidant activity of fortified bread with the highest values when sourdough was the leavening agent. The capacity of hemp flour to deliver biologically active compounds and flavors in bread was also investigated by metabolomics analyses. Multivariate analysis of volatile organic compounds with pro-health impact was able to give insights on the impact of hemp seed flour and sourdough fermentation [80].

## 4. Application of Metabolomics for Wheat and Bread Safety Control

The presence of microbiological and chemical contaminants in food is one of the biggest challenges of food processing. In addition, climate change and environmental pollution could also generate contaminants and prejudicially affect the food quality [81]. For this reason, a lot of efforts have been made in all countries and communities in order to create laws and regulations to ensure public health.

Along with the legislation and claims regarding food safety, rapid, sensitive and effective analytical tools are necessary to detect the contaminants, sometimes present in trace amounts. In this context, the application of metabolomics in food safety control has proven its great potential in the evaluation of microbial toxins, allergens, anti-nutritionals, foodborne pathogens, pesticides [53,82].

Even with the great efforts of food processors, due to the microorganisms’ adaptation, microbial-related contaminants affect food safety frequently. Culture-dependent methods for microorganisms’ identification and characterization are now considered a time-consuming, labor-intensive and expensive approach. So, to prevent and control the negative implications of pathogenic microorganisms and their metabolites, omics techniques overstepped the boundaries by gaining applicability in the detection and identification of food pathogens [83]. Chromatography coupled with mass spectrometry (LC-MS, GC-MS) is usually used to quantify pathogens and toxins and for their identification, a comparison with available libraries is carried out [81]. Also, for this type of study matrix laser desorption/ionization mass spectrometry (MALDI-TOF), Fourier transform infra-red spectroscopy (FTIR) and NMR (which could be ^1^H NMR (proton nuclear magnetic resonance) and 2D NMR (two-dimensional nuclear magnetic resonance) were used [84].

Particular genera and species of fungi could contaminate wheat-producing mycotoxins as secondary toxic metabolites. The producing microorganisms belong to the genera of *Aspergillus, Penicillium, Fusarium* and *Alternaria*, while the most common mycotoxins produced are aflatoxins, fumonisins, ochratoxins, zearalenone, α-zearalanol, β-zearalanol, sterigmatocystin, deoxynivalenol and citrinin. Their toxicity is related to some diseases such as kidney cancer, immune suppression, nephrotoxicity, liver diseases and cancer, reproductive system diseases, leukoencephalomalacia, inhibition of cell growth and division, inhibition of protein synthesis, immune suppression, gastrointestinal diseases, and esophageal cancer [83]. After harvest, storing conditions play a crucial role in stopping aflatogenic fungi to produce aflatoxins and assure wheat safety. But wheat contamination with mycotoxins may occur also in the maturation period or during harvesting and transportation [48]. Metabolomics approaches have been successfully applied to monitor wheat safety over the last few years. Using LC-HRMS, Doppler et al. [85] determined deoxynivalenol in wheat cultivars. Also, by using the metabolomics approach (UHPLC-QTOF-MS), Nathanail et al. [86] identified wheat T-2 toxin, HT-2 toxin, and their metabolism products. Phenylpropanoids and derivatives were determined also in wheat cultivars using LC-MS techniques [87].

Deoxynivalenola, is a mycotoxin from trichothecene group produced by *Fusarium* ssp. and is the most common toxin found in crops all over the world. Deoxynivalenola is usually resistant to the milling process and remains in the flour and baking products [88]. The limits authorized by the European Commission for cereal grain is 1250 μg kg^−1^, while for flour, bran, germ and end products the limit is 750 μg kg^−1^ [89,90]. Metabolomic analysis revealed the presence of deoxynivalenol in wheat milling fraction, flour as well as in bakery products. De Dominicis et al. [91] reported its presence in both flour (10–100 mg/kg) and mini-cakes (20 mg/ kg) when ultra-high-pressure liquid chromatography (UHPLC) coupled with an Orbitrap Exactive™ high-resolution mass spectrometer (HRMS) was performed. Savi et al. [88] also reported that wheat bran contained the highest content of 2278 μg/kg deoxynivalenola, followed by wheat flour (1305 μg/ kg) and bread (437 μg/kg).

Regarding aflatoxin contamination, it is related to the presence of several factors implicated in microbial growth such as temperatures (25–37 °C), moisture (80–85%) and acidic values of pH [92]. Metabolomics studies were performed for aflatoxins detection and quantification and revealed that from 178 samples, 18.8% wheat samples and 8.2% crackers samples were detected positive with aflatoxin B1, even if the toxin content was under the European regulation limit for cereals (2 μg/kg) [89]. Also, wheat samples contained the highest level of aflatoxin B1, while in bread and whole bread this toxin was not found [93].

The employment of some xenobiotics (e.g., pesticides, fungicides, nanomaterials) in agriculture is a frequent practice to increase crop yields. However, the residues of these contaminants in food crops constitute a health risk, even if they are not permitted in higher concentrations than the limits. For detection and accurate determination of pesticides several studies proposed UHPLC-Orbitrap-MS/UPLC-MS method to detect pesticides in very low amounts from different food matrices, among which there are cereals, honey, eggs [94]. Mastovska et al. [95] optimized DSI-LVI-GC-TOFMS and UPLCMS/MS to analyze 180 pesticides between cereals grain. The analyzed pesticides were found in wheat extract ranging from 25–250 ng/g. Ultra-high-pressure liquid chromatography (UHPLC) coupled with an Orbitrap Exactive™ high-resolution mass spectrometer (HRMS) was performed by De Dominicis, Commissati, and Suman [91] reporting in wheat flour the presence of some pesticides (dodine, piperonyl butoxide, tebuconazole and methyl pirimiphos) in the of 1–10 mg/ kg range. GC-TOF (gas chromatography-time-of-flight mass spectrometry) analysis was performed on several types of fat-containing dough by Koesukwiwat et al. [96] to determine residues of pesticide. It was reported that the used method provided acceptable results for most pesticides with overall average recoveries between 70 and 120%. UHPLC-ESI–MS/MS (QqQ in MRM mode) was used for the determination of mycotoxins and pesticides from wheat flour by adapting the extraction method, a fact that improved the recoveries of analytes [97].

Metabolomics is viewed nowadays as a powerful technology able to assess unintended metabolites in food and their changes [98]. Within the metabolomics analysis frame, GC-MS, NMR and LC-MS were mainly used in the case of wheat to determine and differentiate GM (genetic modified) varieties from conventional ones, reporting a broad variation in the metabolites profile such as sugars, sugar phosphates, organic acids, fatty acids, polyols, terpenoides. However, when multivariate PCA analysis was performed in order to discriminate between samples it was found that the differences in metabolite profiling were small and rather due to the natural crop variability or to the environmental conditions [98,99,100].

## 5. Conclusions

In bakery science, metabolomics shows its potential to provide crucial information about several aspects of wheat and bread quality clearly. By analyzing the metabolome of wheat, breeders could develop stress-resistant plants and could predict their adaptability. Moreover, if the wheat molecular markers are combined with flours metabolite profile, wheat breeding for bread aroma will play a key role. It is expected that metabolomics approaches will be used extensively in the breadmaking process and high-quality product development by assessing the compounds involved in consumers’ sensorial preferences and select the optimum combination factors to increase health beneficial features. Thus, metabolomics might be considered a future efficient tool to elucidate the sensory, nutritional and safety quality of wheat bread.

## References

[1] Cauvain S.P., Young L.S. (2006). Baked Products: Science, Technology and Practice.

[2] Martínez-Monzó J., García-Segovia P., Albors-Garrigos J. (2013). Trends and innovations in bread, bakery, and pastry. J. Culin. Sci. Technol..

[3] Mitelut A.C., Popa E.E., Popescu P.A., Popa M.E., Galanakis C.M. (2021). Chapter 7—Trends of innovation in bread and bakery production. Trends in Wheat and Bread Making.

[4] Cifuentes A. (2012). Food analysis in the postgenomic era. Foodomics. Electrophor..

[5] Alvarez-Rivera G., Valdes A., Leon C., Cifuentes A., Barros-Velázquez J. (2021). Chapter 1—Foodomics, fundamentals, state of the art and future trends. Foodomics: Omic Strategies and Applications in Food Science.

[6] Jacobs D.M., Van den Berg M.A., Hall R.D. (2021). Towards superior plant-based foods using metabolomics. Curr. Opin. Biotechnol..

[7] Saia S., Fragasso M., De Vita P., Beleggia R. (2019). Metabolomics provides valuable insight for the study of durum wheat: A review. J. Agric. Food Chem..

[8] Paul A., Harrington P.B. (2021). Chemometric applications in metabolomic studies using chromatography-mass spectrometry. TrAC Trends Anal. Chem..

[9] Villate A., San Nicolas M., Gallastegi M., Aulas P.A., Olivares M., Usobiaga A., Etxebarria N., Aizpurua-Olaizola O. (2020). Review: Metabolomics as a prediction tool for plants performance under environmental stress. Plant Sci..

[10] Cevallos-Cevallos J.M., Reyes-De-Corcuera J.I., Etxeberria E., Danyluk M.D., Rodrick G.E. (2009). Metabolomic analysis in food science: A review. Trends Food Sci. Technol..

[11] Reisdorph N.A., Walmsley S., Reisdorph R. (2020). A perspective and framework for developing sample type specific databases for lc/ms-based clinical metabolomics. Metabolites.

[12] Blaženović I., Kind T., Ji J., Fiehn O. (2018). Software tools and approaches for compound identification of LC-MS/MS data in metabolomics. Metabolites.

[13] Ferri M., Serrazanetti D.I., Tassoni A., Baldissarri M., Gianotti A. (2016). Improving the functional and sensorial profile of cereal-based fermented foods by selecting *Lactobacillus Plantarum* strains via a metabolomics approach. Food Res. Int..

[14] Saa D.L.T., Nissen L., Gianotti A. (2019). Metabolomic approach to study the impact of flour type and fermentation process on volatile profile of bakery products. Food Res. Int..

[15] Longin F., Beck H., Gütler H., Heilig W., Kleinert M., Rapp M., Philipp N., Erban A., Brilhaus D., Mettler-Altmann T. (2020). Aroma and quality of breads baked from old and modern wheat varieties and their prediction from genomic and flour-based metabolite profiles. Food Res. Int..

[16] Guerzoni M.E., Vernocchi P., Ndagijimana M., Gianotti A., Lanciotti R. (2007). Generation of aroma compounds in sourdough: Effects of stress exposure and lactobacilli-yeasts interactions. Food Microbiol..

[17] Quílez J., Ruiz J.A., Romero M.P. (2006). Relationships between sensory flavor evaluation and volatile and nonvolatile compounds in commercial wheat bread type baguette. J. Food Sci..

[18] Ur-Rehman S., Paterson A., Piggott J.R. (2006). Flavour in sourdough breads: A review. Trends Food Sci. Technol..

[19] Vernocchi P., Ndagijimana M., Serrazanetti D., Gianotti A., Vallicelli M., Guerzoni M.E. (2008). Influence of starch addition and dough microstructure on fermentation aroma production by yeasts and lactobacilli. Food Chem..

[20] Brescia M.A., Sgaramella A., Ghelli S., Sacco A. (2003). 1H HR-MAS NMR and isotopic investigation of bread and flour samples produced in southern Italy. J. Sci. Food Agric..

[21] Balestra F., Laghi L., Saa D.T., Gianotti A., Rocculi P., Pinnavaia G.G. (2015). Physico-chemical and metabolomic characterization of KAMUT^®^ Khorasan and durum wheat fermented dough. Food Chem..

[22] Makhoul S., Romano A., Capozzi V., Spano G., Aprea E., Cappellin L., Benozzi E., Scampicchio M., Märk T.D., Gasperi F. (2015). Volatile compound production during the bread-making process: Effect of flour, yeast and their interaction. Food Bioprocess Technol..

[23] Weckx S., Van Kerrebroeck S., Vuyst L.D. (2019). Omics approaches to understand sourdough fermentation processes. Int. J. Food Microbiol..

[24] Singh A. (2020). Tools for metabolomics. Nat. Methods.

[25] Zhen S., Dong K., Deng X., Zhou J., Xu X., Han C., Zhang W., Xu Y., Wang Z., Yan Y. (2016). Dynamic metabolome profiling reveals significant metabolic changes during grain development of bread wheat (*Triticum Aestivum*, L.). J. Sci. Food Agric..

[26] Matros A., Liu G., Hartmann A., Jiang Y., Zhao Y., Wang H., Ebmeyer E., Korzun V., Schachschneider R., Kazman E. (2017). Genome-metabolite associations revealed low heritability, high genetic complexity, and causal relations for leaf metabolites in winter wheat (*Triticum Aestivum*). J. Exp. Bot..

[27] Beleggia R., Rau D., Laidò G., Platani C., Nigro F., Fragasso M., De Vita P., Scossa F., Fernie A.R., Nikoloski Z. (2016). Evolutionary metabolomics reveals domestication-associated changes in tetraploid wheat kernels. Mol. Biol. Evol..

[28] Francki M.G., Hayton S., Gummer J.P.A., Rawlinson C., Trengove R.D. (2016). Metabolomic profiling and genomic analysis of wheat aneuploid lines to identify genes controlling biochemical pathways in mature grain. Plant Biotechnol. J..

[29] Iannucci A., Fragasso M., Beleggia R., Nigro F., Papa R. (2017). Evolution of the crop rhizosphere: Impact of domestication on root exudates in tetraploid wheat (*Triticum Turgidum*, L.). Front. Plant Sci..

[30] Biyiklioglu S., Alptekin B., Akpinar B.A., Varella A.C., Hofland M.L., Weaver D.K., Bothner B., Budak H. (2018). A large-scale multiomics analysis of wheat stem solidness and the wheat stem sawfly feeding response, and syntenic associations in barley, brachypodium, and rice. Funct. Integr. Genom..

[31] Bernardo L., Carletti P., Badeck F.W., Rizza F., Morcia C., Ghizzoni R., Rouphael Y., Colla G., Terzi V., Lucini L. (2019). Metabolomic responses triggered by arbuscular mycorrhiza enhance tolerance to water stress in wheat cultivars. Plant Physiol. Biochem..

[32] Yadav A.K., Carroll A.J., Estavillo G.M., Rebetzke G.J., Pogson B.J. (2019). Wheat drought tolerance in the field is predicted by amino acid responses to glasshouse-imposed drought. J. Exp. Bot..

[33] Righetti L., Rubert J., Galaverna G., Folloni S., Ranieri R., Stranska-Zachariasova M., Hajslov J., Dall’Asta C. (2016). Characterization and discrimination of ancient grains: A metabolomics approach. Int. J. Mol. Sci..

[34] Vicente R., Pérez P., Martínez-Carrasco R., Feil R., Lunn J.E., Watanabe M., Arrivault A., Stitt M., Hoefgen R., Morcuende R. (2016). Metabolic and transcriptional analysis of durum wheat responses to elevated CO_2_ at low and high nitrate supply. Plant Cell Physiol..

[35] Woodrow P., Ciarmiello L.F., Annunziata M.G., Pacifico S., Iannuzzi F., Mirto A., D’Amelia L., Dell’Aversana E., Piccolella S., Fuggi A. (2017). Durum wheat seedling responses to simultaneous high light and salinity involve a fine reconfiguration of amino acids and carbohydrate metabolism. Physiol. Plant..

[36] Kage U., Yogendra K.N., Kushalappa A.C. (2017). TaWRKY70 Transcription factor in wheat *QTL-2DL* regulates downstream metabolite biosynthetic genes to resist *Fusarium Graminearum* infection spread within spike. Sci. Rep..

[37] Righetti L., Rubert J., Galaverna G., Hurkova K., Dall’Asta C., Hajslova J., Stranska-Zachariasova M. (2018). A novel approach based on untargeted lipidomics reveals differences in the lipid pattern among durum and common wheat. Food Chem..

[38] Zhang Y., Ma X.M., Wang X.C., Liu J.H., Huang B.I., Guo X.Y., Xiong S.P., La G.X. (2017). UPLC-QTOF Analysis reveals metabolomic changes in the flag leaf of wheat (*Triticum Aestivum*, L.) under low-nitrogen stress. Plant Physiol. Biochem..

[39] Thomason K., Babar M.A., Erickson J.E., Mulvaney M., Beecher C., MacDonald G. (2018). Comparative physiological and metabolomics analysis of wheat (*Triticum Aestivum*, L.) following post-anthesis heat stress. PLoS ONE.

[40] Guo R., Shi L.X., Jiao Y., Li M.X., Zhong X.L., Gu F.X., Liu X., Xia X., Li H.R. (2018). Metabolic responses to drought stress in the tissues of drought-tolerant and drought-sensitive wheat genotype seedlings. AoB Plants.

[41] Vergara-Diaz O., Vatter T., Vicente R., Obata T., Nieto-Taladriz M.T., Aparicio N., Kefauver S.C., Fernie A., Araus J.L. (2020). Metabolome profiling supports the key role of the spike in wheat yield performance. Cells.

[42] Guo X., Xin Z., Yang T., Ma X., Zhang Y., Wang Z., Ren Y., Lin T. (2020). Metabolomics response for drought stress tolerance in chinese wheat genotypes (*Triticum Aestivum*). Plants.

[43] Shewry P.R., Corol D.I., Jones J.D., Beale M.H., Ward J.L. (2017). Defining genetic and chemical diversity in wheat grain by 1H-NMR spectroscopy of polar metabolites. Mol. Nutr. Food Res..

[44] Itam M., Mega R., Tadano S., Abdelrahman M., Matsunaga S., Yamasaki Y., Akashi K., Tsujimoto H. (2020). Metabolic and physiological responses to progressive drought stress in bread wheat. Sci. Rep..

[45] Narayanan S., Prasad P.V.V., Ruth W. (2016). Wheat leaf lipids during heat stress: II. Lipids experiencing coordinated metabolism are detected by analysis of lipid co-occurrence. Plant Cell Env..

[46] Dal C., Barion C.G., Visioli G., Mattarozzi M., Mosca G., Vamerali T. (2017). Increased root growth and nitrogen accumulation in common wheat following PGPR inoculation: Assessment of plant-microbe interactions by ESEM. Agric. Ecosyst. Environ..

[47] Ziegler J.U., Steingass C.B., Longin C.F.H., Würschum T., Carle R., Schweiggert R.M. (2015). Alkylresorcinol composition allows the differentiation of *Triticum* spp. having different degrees of ploidy. J. Cereal Sci..

[48] Varzakas T. (2016). Quality and safety aspects of cereals (wheat) and their products. Crit. Rev. Food Sci. Nutr..

[49] Cauvain S. (2012). Breadmaking Improving Quality.

[50] Gänzle M.G. (2014). Enzymatic and bacterial conversions during sourdough fermentation. Food Microbiol..

[51] Verni M., Rizzello C.G., Coda R. (2019). Fermentation biotechnology applied to cereal industry by-products: Nutritional and functional insights. Front. Nutr..

[52] Fernández-Peláez J., Paesani C., Gómez M. (2020). Sourdough technology as a tool for the development of healthier grain-based products: An update. Agronomy.

[53] Peñas E., Diana M., Frias J., Quílez J., Martínez-Villaluenga C. (2015). A multistrategic approach in the development of sourdough bread targeted towards blood pressure reduction. Plant Foods Hum. Nutr..

[54] Li K.J., Brouwer-Brolsma E.M., Burton-Pimentel K.J., Vergères G., Feskens E.J.M. (2021). A Systematic review to identify biomarkers of intake for fermented food products. Genes Nutr..

[55] Chiş M., Păucean A., Man S., Vodnar D., Teleky B.-E., Pop C., Stan L., Borsai O., Kadar C., Urcan A. (2020). Quinoa sourdough fermented with *Lactobacillus Plantarum* ATCC 8014 designed for gluten-free muffins—A powerful tool to enhance bioactive compounds. Appl. Sci..

[56] Hansen A., Schieberle P. (2005). Generation of aroma compounds during sourdough fermentation: Applied and fundamental aspects. Trends Food Sci. Technol..

[57] Pizarro F., Franco F. (2017). Volatile organic compounds at early stages of sourdough preparation via static headspace and GC/MS analysis. Curr. Res. Nutr. Food Sci..

[58] Nakamura T., Tomita S., Saito K. (2018). Metabolite profiling in dough during fermentation. Food Sci. Technol. Res..

[59] Colosimo R., Gabriele M., Cifelli M., Longo V., Domenici V., Pucci L. (2020). The effect of sourdough fermentation on *Triticum Dicoccum* from Garfagnana: 1H NMR characterization and analysis of the antioxidant activity. Food Chem..

[60] Koca N., Karaman Ș. (2015). The effects of plant growth regulators and L-phenylalanine on phenolic compounds of sweet basil. Food Chem..

[61] Arendt E.K., Moroni A., Zannini E. (2011). Medical nutrition therapy: Use of sourdough lactic acid bacteria as a cell factory for delivering functional biomolecules and food ingredients in gluten free bread. Microb. Cell Factories.

[62] Chiș M.S., Păucean A., Stan L., Suharoschi R., Socaci S.A., Man S.M., Pop C.R., Muste S. (2019). Impact of protein metabolic conversion and volatile derivatives on gluten-free muffins made with quinoa sourdough. CYTA J. Food.

[63] Lamberts L., Joye I.J., Beliën T., Delcour J.A. (2012). Dynamics of γ-aminobutyric acid in wheat flour bread making. Food Chem..

[64] Karaman K., Sagdic O., Durak M.Z. (2018). Use of phytase active yeasts and lactic acid bacteria isolated from sourdough in the production of whole wheat bread. LWT Food Sci. Technol..

[65] Fekri A., Torbati M., Khosrowshahi A.Y., Shamloo H.B., Azadmard-Damirchi S. (2020). Functional effects of phytate-degrading, probiotic lactic acid bacteria and yeast strains isolated from Iranian traditional sourdough on the technological and nutritional properties of whole wheat bread. Food Chem..

[66] Salmeron I., Fuciños P., Charalampopoulos D., Pandiella S.S. (2009). Volatile compounds produced by the probiotic strain lactobacillus plantarum NCIMB 8826 in cereal-based substrates. Food Chem..

[67] Koistinen V.M., Mattila O., Katina K., Poutanen K., Aura A.M., Hanhineva K. (2018). Metabolic profiling of sourdough fermented wheat and rye bread. Sci. Rep..

[68] Monirujjaman M., Ferdouse A. (2014). Metabolic and physiological roles of branched-chain amino acids. Adv. Mol. Biol..

[69] Holeček M. (2018). Branched-chain amino acids in health and disease: Metabolism, alterations in blood plasma, and as supplements. Nutr. Metab..

[70] Esfandi R., Walters M.E., Tsopmo A. (2019). Antioxidant properties and potential mechanisms of hydrolyzed proteins and peptides from cereals. Heliyon.

[71] Călinoiu L.F., Vodnar D.C. (2018). Whole grains and phenolic acids: A review on bioactivity, functionality, health benefits and bioavailability. Nutrients.

[72] Galli V., Venturi M., Guerrini S., Blandino M., Luti S., Pazzagli L., Granchi Lisa (2020). Antioxidant properties of sourdoughs made with whole grain flours of hull-less barley or conventional and pigmented wheat and by selected lactobacilli strains. Foods.

[73] Di Renzo T., Reale A., Boscaino F., Messia M.C. (2018). Flavoring production in Kamut^®^, quinoa and wheat doughs fermented by lactobacillus paracasei, lactobacillus plantarum, and lactobacillus brevis: A SPME-GC/MS study. Front. Microbiol..

[74] Struyf N., Van der Maelen E., Hemdane S., Verspreet J., Verstrepen K.J., Courtin C.M. (2017). Bread dough and baker’s yeast: An uplifting synergy. Compr. Rev. Food Sci. Food Saf..

[75] Yan B., Sadiq F.A., Cai Y., Fan D., Zhang H., Zhao J., Chen W. (2019). Identification of key aroma compounds in type I sourdough-based Chinese steamed bread: Application of untargeted metabolomics analysisp. Int. J. Mol. Sci..

[76] Zhang G., Sun Y., Sadiq F.A., Sakandar H.A., He G. (2018). Evaluation of the effect of saccharomyces cerevisiae on fermentation characteristics and volatile compounds of sourdough. J. Food Sci. Technol..

[77] Erban A., Fehrle I., Martinez-Seidel F., Brigante F., Más A.L., Baroni V., Wunderlin D., Kopka J. (2019). Discovery of food identity markers by metabolomics and machine learning technology. Sci. Rep..

[78] Rocchetti G., Rizzi C., Cervini M., Rainero G., Bianchi F., Giuberti G., Lucini L., Simonato B. (2021). Impact of grape pomace powder on the phenolic bioaccessibility and on in vitro starch digestibility of wheat based bread. Foods.

[79] Taglieri I., Sanmartin C., Venturi F., Macaluso M., Bianchi A., Sgherri C., Quartacci M.F., De Leo M., Pistelli L., Palla F. (2021). Bread fortified with cooked purple potato flour and citrus albedo: An evaluation of its compositional and sensorial properties. Foods.

[80] Nissen L., Bordoni A., Gianotti A. (2020). Shift of volatile organic compounds (VOCs) in gluten-free hemp-enriched sourdough bread: A metabolomic approach. Nutrients.

[81] Pinu F.R. (2015). Metabolomics—The new frontier in food safety and quality research. Food Res. Int..

[82] Li S., Tian Y., Jiang P., Lin Y., Liu X., Yang H. (2021). Recent advances in the application of metabolomics for food safety control and food quality analyses. Crit. Rev. Food Sci. Nutr..

[83] Oyedeji A.B., Green E., Adebiyi J.A., Ogundele O.M., Gbashi S., Adefisoye M.A., Oyeyinka S.A., Adebo O.A. (2021). Metabolomic approaches for the determination of metabolites from pathogenic microorganisms: A review. Food Res. Int..

[84] Zhao X., Chen L., Wu J.E., He Y., Yang H. (2020). Elucidating antimicrobial mechanism of nisin and grape seed extract against *Listeria monocytogenes* in broth and on shrimp through NMR-based metabolomics approach. Int. J. Food Microbiol..

[85] Dropler M., Kluger B., Bueschl C., Steiner B., Buerstmayr H., Lemmens M., Krska R., Adam G., Schuhmacher R. (2019). Stable isotope-assisted plant metabolomics: Investigation of phenylalanine-related metabolic response in wheat upon treatment with the *Fusarium* virulence factor deoxynivalenol. Front. Plant Sci..

[86] Nathanail A.V., Syvähuoko J., Malachová A., Jestoi M., Varga E., Michlmayr H., Adam G., Sieviläinen E., Berthiller F., Peltonen K. (2015). Simultaneous determination of major type A and B trichothecenes, zearalenone and certain modified metabolites in Finnish cereal grains with a novel liquid chromatography-tandem mass spectrometric method. Anal. Bioanal. Chem..

[87] Gunnaiah R., Kushalappa A.C. (2014). Metabolomics deciphers the host resistance mechanisms in wheat cultivar sumai-3, against trichothecene producing and non-producing isolates of *Fusarium Graminearum*. Plant Physiol. Biochem..

[88] Savi G.D., Piacentini K.C., Tibola C.S., Santos K., Maria G.S., Scussel V.M. (2016). Deoxynivalenol in the wheat milling process and wheat-based products and daily intake estimates for the southern Brazilian population. Food Control.

[89] (2006). Commission Regulation (EC), No 1881/2006 Setting Maximum Levels for Certain Contaminants in Foodstuffs.

[90] European Commission (2007). European Commission Regulation No1126/2007 amending Regulation No1881/2006 setting maximum levels for certain contaminants sin foodstuffs as regards Fusarium toxins in maize and maize products. Off. J. Eur. Union L.

[91] De Dominicis E., Commissati I., Suman M. (2012). Targeted screening of pesticides, veterinary drugs and mycotoxins in bakery ingredients and food commodities by liquid chromatography-high-resolution single-stage orbitrap Mass Spectrometry. J. Mass Spectrom..

[92] Nazhand A., Durazzo A., Lucarini M., Souto E.B., Santini A. (2020). Characteristics, occurrence, detection and detoxification of aflatoxins in foods and feeds. Foods.

[93] Zhao Y., Wang Q., Huang J., Ma L., Chen Z., Wang F. (2018). Aflatoxin B1 and sterigmatocystin in wheat and wheat products from supermarkets in China. Food Addit. Contam. Part B Surveill..

[94] Syed A.A., Nazir S., Adnan M., Azad Z.R.A.A. (2020). UPLC-MS: An emerging novel technology and its application in food safety.

[95] Mastovska K., Dorweiler K.J., Lehotay S.J., Wegscheid J.S., Szpylka K.A. (2010). Pesticide multiresidue analysis in cereal grains using modified QuEChERS method combined with automated direct sample introduction GC-TOFMS and UPLC-MS/MS techniques. J. Agric. Food Chem..

[96] Koesukwiwat U., Lehotay S.J., Mastovska K., Dorweiler K.J., Leepipatpiboon N. (2010). Extension of the QuEChERS method for pesticide residues in cereals to flaxseeds, peanuts, and doughs. J. Agric. Food Chem..

[97] Lacina O., Zachariasova M., Urbanova J., Vaclavikova M., Cajka T., Hajslova J. (2012). Critical assessment of extraction methods for the simultaneous determination of pesticide residues and mycotoxins in fruits, cereals, spices and oil seeds employing ultra-high performance liquid chromatography-tandem mass spectrometry. J. Chromatogr. A.

[98] Shepherd L.V., Fraser P., Stewart D. (2011). Metabolomics: A second-generation platform for crop and food analysis. Bioanalysis.

[99] Baker J.M., Hawkins N.D., Ward J.L., Lovegrove A., Napier J.A., Shewry P.R., Beale M.H. (2006). A Metabolomic study of substantial equivalence of field-grown genetically modified wheat. Plant Biotechnol. J..

[100] Ward J.L., Shewry P.R. (2010). Future prospects for the analysis of bioactive components in cereal grain. Cereal Foods World.

